# Childhood Family Violence and Tobacco, E-Cigarette, and Alcohol Use Among Adolescents: A Large School-Based Study in China

**DOI:** 10.3390/healthcare14131814

**Published:** 2026-06-23

**Authors:** Zhicheng Zhen, Yiming Liu, Yue Gao, Jing An, Hossein Zare

**Affiliations:** 1 Department of Health Policy and Management, Bloomberg School of Public Health, Johns Hopkins University, Baltimore, MD 21202, USA; 2 Institute for Hospital Management, Tsinghua University, Shenzhen Campus, Shenzhen 518071, China; 3 Global Health Research Center, Duke Kunshan University, Kunshan 215316, China; yiming.liu@dukekunshan.edu.cn; 4 Beijing Anding Hospital, Beijing 100088, China; 5 Beijing Huilongguan Hospital, Beijing 100096, China; 6 HuilongGuan Clinical Medical School, Peking University, Beijing 100871, China; 7 WHO Collaborating Center for Research and Training in Suicide Prevention, Beijing 100096, China

**Keywords:** childhood family violence, tobacco use, e-cigarette, alcohol use, adolescents

## Abstract

**Background:** Adolescent tobacco, e-cigarette, and alcohol use are important public health concerns in China. However, the associations of specific types and cumulative exposure to childhood family violence with different substance use outcomes remain insufficiently understood. This study examined these associations among Chinese adolescents. **Methods:** We analyzed data from a cross-sectional school-based survey of 41,146 students aged 10–19 years conducted from October 2022 to March 2023 in a mountainous city in western Guangdong Province, China. Childhood family violence was assessed using the validated Chinese Family Violence Questionnaire and a cumulative exposure index. Descriptive analyses and logistic regression models were conducted, with adjustment for demographic and environmental factors. **Results:** The mean age of participants was 14.8 years, and 51.7% were female. Overall, 25.1% of adolescents reported at least one type of childhood family violence. Verbal insults (18.6%) and emotional neglect (16.3%) were the most frequently reported types and were consistently associated with tobacco, e-cigarette, and alcohol use (adjusted odds ratios [ORs] = 1.4–1.5, *p* < 0.001). A cumulative exposure pattern was also observed. Compared with adolescents reporting no childhood family violence, those exposed to three or more types had higher odds of tobacco use (OR = 3.81; 95% CI: 3.42–4.23), e-cigarette use (OR = 3.90; 95% CI: 3.39–4.48), and alcohol use (OR = 3.95; 95% CI: 3.59–4.35). Peer smoking and access to tobacco products were also significantly associated with substance use. **Conclusions:** Childhood family violence, particularly verbal insults and emotional neglect, was associated with adolescent tobacco, e-cigarette, and alcohol use. The findings highlight the importance of considering emotional maltreatment, cumulative adversity, peer influences, and access to tobacco products in future prevention research and practice.

## 1. Introduction

Adolescence is a critical developmental period when experimentation with tobacco, e-cigarettes, and alcohol may be associated with long-term health and social consequences. Early initiation of these substances is associated with persistent addiction, chronic disease, impaired neurocognitive development, and social disadvantage in adulthood [1,2,3]. Identifying upstream determinants of these behaviors is essential for prevention strategies that move beyond individual-level education to address the broader ecology of adolescent risk.

Childhood family violence may be associated with a higher likelihood of substance use during adolescence. Such violence can take multiple forms, including physical violence, verbal abuse, emotional neglect, sexual violence, and physical neglect. These experiences may be associated with altered stress regulation, difficulties in emotion regulation and reward processing, and greater reliance on maladaptive coping strategies [1,2,4,5]. Longitudinal data from China have linked sexual abuse and neglect to persistent smoking and drinking trajectories across adolescence [1]. Studies from Puerto Rico and Brazil have also associated household violence with earlier initiation and later problematic alcohol use [2,4]. Evidence from Mexico and Uganda similarly suggests that adolescents exposed to child maltreatment or domestic violence may be more likely to report smoking, vaping, and hazardous drinking [4,5,6]. Beyond single exposures, research on poly-victimization suggests that the accumulation of multiple adversities is associated with progressively higher risks of substance initiation and escalation [7,8,9].

Adolescent substance use is also shaped by broader social and environmental contexts. Peer influence has consistently been an important factor in smoking and drinking across settings; adolescents whose close friends use tobacco or alcohol are substantially more likely to initiate and sustain use themselves [10,11]. Access to tobacco and e-cigarettes, through weak retail enforcement and informal channels, has been shown to accelerate uptake and dual use [3,12]. Parental factors, including low educational attainment, harsh or inconsistent parenting, and parental smoking or alcohol misuse, may contribute to a more adverse home environment. Parental substance use may also model substance-related behaviors, thereby compounding adolescent risk [13,14,15]. Socioeconomic disadvantage compounds these risks by elevating family stress, limiting supportive resources, and weakening regulatory protections [16,17]. Together, these findings underscore that adolescent substance use is shaped by a complex interplay between individual vulnerability, early family adversity, and modifiable social determinants.

Despite growing international evidence, population-level data from China remain limited. Most studies have focused on broad categories of maltreatment without differentiating specific forms of childhood family violence, despite evidence that emotional neglect and verbal abuse may be particularly potent in shaping adolescent risk-taking [1,7,8]. The dose–response relationship between cumulative violence exposure and multiple substance types, including the rapidly expanding use of e-cigarettes, is poorly characterized. Moreover, although China is experiencing rapid increases in adolescent e-cigarette use and persistent concerns about smoking and underage drinking [18], few studies have incorporated environmental determinants, such as peer smoking, teacher modeling, and access to tobacco products, alongside family-level risks. Understanding how these domains intersect is crucial for designing prevention programs that are culturally and contextually relevant.

China’s sociocultural and policy context also adds urgency to this inquiry. Although national efforts to restrict youth access to tobacco and e-cigarettes have strengthened, enforcement remains uneven, particularly in less economically developed regions [19]. The study was conducted in a mountainous city in western Guangdong Province, China, with a population of approximately 2.4 million people and a relatively high proportion of left-behind children whose parents have migrated for work. These structural vulnerabilities may both increase the risk of childhood family violence and limit adolescents’ access to timely psychological support or prevention programs.

Against this backdrop, the present study aimed to examine the associations between specific types and cumulative exposure to childhood family violence and lifetime substance use, including tobacco, e-cigarettes, and alcohol, among Chinese adolescents. Critically, we accounted for a broad range of potential confounders and co-occurring risks, including demographic factors (age, gender, residence, one-child status), socioeconomic status, parental education, parental smoking, teacher smoking, peer smoking, and access to tobacco products. By jointly considering family adversity and environmental factors, this study provides context-specific evidence to inform future prevention research and practice.

## 2. Methods

### 2.1. Study Population (Participants)

The cross-sectional survey was conducted from October 2022 through March 2023 in a mountainous city in western Guangdong Province, China. Electronic informed consent was obtained from both students and their parents or legal guardians. Students who voluntarily agreed to participate then registered and completed the self-administered questionnaire individually in their classrooms during regular school hours through a structured online data collection platform developed by the research team. They used either school computers or mobile phones provided by the research team. At least one trained teacher was present on-site to maintain discipline and order and, when necessary, to respond to students’ questions about the questionnaire completion process. Teachers did not review or access students’ responses. To protect confidentiality, survey data were stored only in the research team’s secure platform database and were de-identified before analysis. These confidentiality procedures were explained to participants during the informed consent process.

This study was conducted as a city-wide school-based survey intended to cover all eligible primary, middle, and high schools in the city. All eligible schools were invited to participate, and eligible students within each participating school were invited to complete the questionnaire voluntarily. A total of 50,222 students were recruited into this study, of whom 46,233 completed all questionnaires, resulting in a response rate of 92.06%. To ensure that the analytic sample focused on adolescents, we excluded 5087 participants who were younger than 10 or older than 19 years, resulting in a final analytic sample of 41,146 students (mean age = 14.81, SD = 1.63; female = 48.3%). No sampling weights or additional stratification procedures were applied. Residence and school level were included as covariates in the regression models to account for potential differences across rural and urban settings and across school levels. The study was approved by the Institutional Review Board of Tsinghua University (No. 20220102).

### 2.2. Measures

*Tobacco and e-cigarette use* were assessed using the Chinese version of the Global Youth Tobacco Survey (GYTS) [20], a school-based instrument developed by the World Health Organization and the U.S. Centers for Disease Control and Prevention and validated for use among Chinese adolescents [21]. Lifetime tobacco and e-cigarette use were coded as binary variables (1 = any lifetime use; 0 = no lifetime use).

*Alcohol use* was assessed using the Alcohol Use Disorders Identification Test (AUDIT), which has demonstrated robust cross-cultural validity in general and excellent validity and reliability for the Chinese translated version specifically [22,23]. The frequency of drinking was dichotomized as 0 (never) and 1 (any alcohol use).

*Childhood family violence* was measured with the Chinese version of the Family Violence Questionnaire, adapted from Bernstein et al. [24] The scale includes five subtypes: physical violence, verbal insults, emotional neglect, sexual violence, and physical neglect. Physical violence refers to experiences within the family, such as being beaten, restrained, or subjected to restrictions on personal freedom that caused fear. Verbal insults refer to being insulted or verbally abused within the family in ways that cause feelings of humiliation or fear. Emotional neglect refers to feeling disregarded, uncared for, or treated with indifference within the family to the extent that it results in feelings of worthlessness. Sexual violence refers to molestation or sexual assault by a family member or relative under coercion or in circumstances in which the adolescent was unable to resist. Physical neglect refers to experiences such as having no one to provide care, insufficient food, having to wear dirty clothing, or not being taken to see a doctor when ill. Each item is rated from 0 (never) to 4 (almost every day), with higher scores indicating greater exposure.

For the demographic variables, we included: Age (years, continuous), Gender (0 = Female, 1 = Male), Grade (0= Primary school and Junior high school, 1= High school), Residence (0 = Rural 1 = Urban), One-child status (1 = Yes, 0 = No), Father’s/Mother’s education level (0= Less than high school, 1 = High school graduate, 2 = College or above), and Socioeconomic status (SES). SES was assessed by asking participants to rate their family’s position in society based on income, education, and occupational respect on a scale of 1 to 10, where 1 represents the lowest class, and 10 represents the highest class [25]. Additionally, access to tobacco or e-cigarettes (1 = Yes, 0 = No), parental tobacco use (1 = Yes, 0 = No), teacher tobacco use (1 = Yes, 0 = No), and peer tobacco use (1 = Yes, 0 = No).

### 2.3. Statistical Analysis

We first conducted descriptive analyses for each type of childhood family violence (physical violence, verbal insult, emotional neglect, sexual violence, and physical neglect). Continuous variables were summarized as means with standard deviations (SD), and categorical variables as frequencies with percentages.

To examine the associations between childhood family violence and adolescent substance use, we fitted a series of logistic regression models [26]. Fully adjusted models controlled for demographic covariates (age, gender, residence, one-child status, socioeconomic status, and parental education level) and environmental risk factors (access to tobacco products, parental tobacco use, teacher tobacco use, and peer tobacco use). Model 1 was an unadjusted model examining the association between childhood family violence and substance use. Model 2 additionally adjusted for demographic characteristics, including age, gender, grade, residence, one-child status, socioeconomic status, and parental education level. Model 3 further adjusted for environmental tobacco-related factors, including access to tobacco products and tobacco use by parents, teachers, and peers. Adjusted odds ratios (ORs) and 95% confidence intervals (CIs) were reported for each type of childhood family violence. For the main analysis, childhood family violence was entered as a continuous composite score (range 0–20) by summing the item responses across the five violence subtypes (each rated 0–4). For subtype-specific analyses, each violence subtype was entered as its original continuous score (range 0–4).

To assess the cumulative impact of childhood family violence, an additive exposure index was created by summing the number of distinct family violence types experienced during childhood, with each subtype considered present if the corresponding item score was greater than 0. This count was then categorized into 0, 1, 2, and 3 or more types, with 0 serving as the reference group. Logistic regression models were then used to evaluate the dose–response relationship between cumulative exposure and each substance use outcome, with adolescents reporting no childhood family violence serving as the reference group. Model fit was evaluated using the Akaike Information Criterion (AIC) and Bayesian Information Criterion (BIC), with lower values indicating better fit [27]. All statistical tests were two-sided, and a *p* value < 0.05 was considered statistically significant. All analyses were performed using R (version 4.1.0) [28].

## 3. Results

### 3.1. Descriptive Statistics and Prevalence of Childhood Family Violence and Substance Use

Table 1 presents the characteristics of the analytic sample, which included 41,146 adolescents aged 10–19 years (mean age = 14.8, SD = 1.6). The gender distribution was relatively balanced, with males accounting for 48.3% (n = 19,863). Most participants were in primary school or junior high school (64.8%, n = 26,663), and the remainder were in high school (35.2%, n = 14,483). More than half of the participants resided in urban areas (53.5%, n = 22,025), while the remainder lived in rural regions (46.5%, n = 19,121).

Regarding family structure, a large majority (91.1%, n = 37,471) were from non-only-child households. Parental education levels were predominantly below high school, with 68.1% reporting less than high school education (n = 28,024), 22.1% reporting high school graduation (n = 9103), and 9.8% reporting college or above (n = 4019). The mean self-reported socioeconomic status score was 5.03 (SD = 1.84).

As shown in Table 2, 25.1% of adolescents (n = 10,316) reported experiencing some form of family violence during childhood. Verbal insults (18.6%) and emotional neglect (16.3%) were the most reported types. Physical violence was reported by 7.4%, and sexual violence by 2.0%. In terms of cumulative exposure, 74.9% reported no experiences of childhood family violence, 11.7% reported exposure to one type, 7.6% to two types, 5.8% to three or more types of childhood family violence.

Table 3 presents the substance use patterns and related environmental risk factors. Overall, 8115 adolescents (19.7%) reported lifetime use of at least one of the three substances. Alcohol use was the most prevalent substance use outcome (13.7%), followed by tobacco use (10.4%) and e-cigarette use (4.7%). Regarding environmental risk factors, 5.8% of participants reported access to tobacco products. Parental tobacco use showed a pronounced gender disparity, with paternal smoking reported by 58.4% of participants, compared with only 1.4% for maternal smoking. Exposure to tobacco use by friends and teachers was relatively low, at 1.1% and 0.8%, respectively.

### 3.2. Multiple Logistic Regression Analysis

**Association between childhood family violence and tobacco use:** Table 4 reports the results of the logistic regression models, as presented family violence was consistently associated with higher odds of tobacco use. The association was evident in the unadjusted model (Model 1: OR = 1.11, 95% CI 1.10–1.12), persisted after adjustment for demographic covariates (Model 2: OR = 1.11, 95% CI 1.10–1.13), and was slightly attenuated but remained significant after further adjustment for tobacco-related environmental factors (Model 3: OR = 1.09, 95% CI 1.08–1.10). Model fit improved with sequential adjustment. In the fully adjusted model, male students had higher odds of tobacco use than female students (OR = 1.50, 95% CI 1.40–1.61), and older age was also associated with increased odds (OR = 1.10, 95% CI 1.06–1.13). Higher socioeconomic status was associated with lower odds (OR = 0.94, 95% CI 0.92–0.96). Compared with adolescents whose parents had less than a high school education, parental education was not significantly associated with tobacco use (high school graduate: OR = 0.93, 95% CI 0.85–1.01; college or above: OR = 0.98, 95% CI 0.90–1.05). Among environmental factors, access to tobacco products (OR = 4.16, 95% CI 3.77–4.59) and friend smoking (OR = 4.15, 95% CI 3.81–4.52) showed the strongest associations; father smoking and teacher smoking were also significant.

**Association between Childhood family violence and electronic cigarette use: Childhood** family violence was consistently associated with higher odds of electronic cigarette use (Table 5). The association was observed in the unadjusted model (Model 1: OR = 1.11, 95% CI 1.10–1.13), persisted after adjusting for demographic covariates (Model 2: OR = 1.11, 95% CI 1.10–1.13), and was slightly attenuated but remained significant after further adjustment for tobacco-related environmental factors (Model 3: OR = 1.09, 95% CI 1.07–1.10). Model fit improved with sequential adjustment. In the fully adjusted model, male students had higher odds of e-cigarette use than female students (OR = 1.66, 95% CI 1.50–1.84), and urban residence was also associated with higher odds (OR = 1.20, 95% CI 1.08–1.33). Among environmental factors, friend smoking and access to tobacco products showed the strongest associations, followed by father smoking and mother smoking.

**Association between Childhood family violence and alcohol use: Childhood** family violence was also associated with higher odds of alcohol use (Table 6). The association was observed in the unadjusted model (Model 1: OR = 1.13, 95% CI 1.11–1.14), remained unchanged after adjusting for demographic covariates (Model 2: OR = 1.13, 95% CI 1.12–1.14), and was slightly attenuated but still significant after further adjustment for tobacco-related environmental factors (Model 3: OR = 1.11, 95% CI 1.10–1.12). Model fit improved with sequential adjustment. In the fully adjusted model, male students had higher odds of alcohol use than female students (OR = 1.13, 95% CI 1.06–1.20), and older age was also associated with increased odds (OR = 1.12, 95% CI 1.08–1.15). Grade (OR = 1.12, 95% CI 1.01–1.23) and urban residence (OR = 1.28, 95% CI 1.20–1.36) were associated with higher odds of alcohol use, whereas higher socioeconomic status was associated with lower odds (OR = 0.95, 95% CI 0.94–0.97). Compared with adolescents whose parents had less than a high school education, having parents who were high school graduates was associated with higher odds of alcohol use (OR = 1.12, 95% CI 1.04–1.20), while college or above was not (OR = 0.96, 95% CI 0.90–1.03). Among environmental factors, friend smoking and access to tobacco products showed the strongest associations; father smoking and teacher smoking were also significant.

**Substance Use among Adolescents with Childhood Family Violence: Table 7** shows the results for each type of childhood family violence; among them, verbal insults and emotional neglect exhibited the most consistent associations. Specifically, verbal insults were associated with higher odds of tobacco use (OR = 1.44; 95% CI: 1.39–1.50), e-cigarette use (OR = 1.43; 95% CI: 1.24–1.62), and alcohol use (OR = 1.51; 95% CI: 1.46–1.57).

Emotional neglect was similarly associated with increased odds of tobacco use (OR = 1.43; 95% CI: 1.37–1.49), e-cigarette use (OR = 1.38; 95% CI: 1.30–1.46), and alcohol use (OR = 1.52; 95% CI: 1.47–1.58). Physical violence also showed consistent associations with tobacco use (OR = 1.35; 95% CI: 1.27–1.43), e-cigarette use (OR = 1.34; 95% CI: 1.24–1.46), and alcohol use (OR = 1.42; 95% CI: 1.35–1.50). Physical neglect, although less strongly associated, remained a significant risk factor, with ORs ranging from 1.17 to 1.26 across the three outcomes. Sexual violence was significantly associated with e-cigarette use (OR = 1.19; 95% CI: 1.08–1.31) and alcohol use (OR = 1.14; 95% CI: 1.06–1.22), but its association with tobacco use did not reach statistical significance (OR = 1.07; 95% CI: 0.99–1.16; *p* = 0.086).

Across each childhood family violence subtype, environmental factors were still significantly associated with adolescent substance use in the fully adjusted model. Peer smoking exposure demonstrated the strongest associations across all outcomes, with adjusted odds ratios (ORs) ranging from 3.90 to 5.79. Access to tobacco products was also a powerful factor (ORs = 2.45–4.78). In addition, paternal, maternal, and teacher smoking were each significantly and positively associated with adolescent smoking behaviors, although the magnitude of these associations varied by source.

To further examine the cumulative impact of childhood family violence exposure, we constructed an additive risk model incorporating the total number of distinct family violence types experienced during childhood. As shown in Table 8, there was a clear dose–response relationship between the number of family violence exposures and the odds of substance use, with higher cumulative exposure associated with substantially elevated risks for tobacco, electronic cigarette, and alcohol use.

Compared with adolescents who reported no exposure to childhood family violence, those exposed to one type already demonstrated significantly increased odds of tobacco use (OR = 2.26; 95% CI: 2.06–2.47), electronic cigarette use (OR = 2.07; 95% CI: 1.81–2.36), and alcohol use (OR = 2.34; 95% CI: 2.16–2.54). The risk further increased with each additional type of violence. Adolescents exposed to two types of family violence had nearly threefold higher odds of tobacco use (OR = 2.95; 95% CI: 2.66–3.27), e-cigarette use (OR = 2.90; 95% CI: 2.51–3.35), and alcohol use (OR = 3.18; 95% CI: 2.90–3.48). The highest odds were observed among those exposed to three or more types of family violence, with significantly increased odds of tobacco use, electronic cigarette use, and alcohol use, relative to adolescents with no family violence exposure.

## 4. Discussion

### 4.1. Emotional Maltreatment and Cumulative Childhood Family Violence

This study found that childhood exposure to family violence, particularly verbal insults and emotional neglect, was significantly and dose-dependently associated with tobacco, e-cigarette, and alcohol use after adjustment for socioeconomic and environmental factors. These findings are consistent with decades of work on the long-term health impact of adverse childhood experiences (ACEs) [29,30] and extend this evidence to the Chinese context, where population-level data on maltreatment and adolescent risk behaviors remain scarce. While previous ACE studies have often emphasized physical and sexual abuse as important correlates of adolescent substance use [31], recent evidence from western cohorts highlights the critical role of emotional maltreatment, including verbal abuse and neglect, in shaping later nicotine and alcohol dependence [32]. Our data suggest that this pattern may also be relevant in non-Western sociocultural contexts, where emotional aggression and neglect may be more normalized yet equally damaging.

The cumulative risk gradient observed in this study is consistent with the growing literature on poly-victimization, which has reported stronger associations with adverse outcomes among individuals exposed to multiple adversities [33,34]. Adolescents reporting three or more violence types faced three- to fourfold higher odds of smoking or drinking than their non-exposed peers. This may reflect a more severe and chronic family stress environment in which repeated exposure may be associated with poorer self-regulation and greater reliance on short-term coping strategies, thereby elevating susceptibility to substance use. These findings also highlight the value of considering cumulative violence exposure in risk stratification, as focusing on single forms of childhood family violence may underestimate risk among adolescents experiencing multiple co-occurring violence types.

### 4.2. Potential Neurobiological, Psychological, and Social Pathways

The observed associations may reflect interacting neurobiological, psychological, and social processes rather than any single factor. Prior research suggests that chronic exposure to childhood family violence may be associated with altered hypothalamic–pituitary–adrenal axis functioning and reward-related processing, which could contribute to vulnerability to nicotine and alcohol use [35,36]. Emotional neglect and verbal hostility may be associated with difficulties in emotion regulation and self-concept development, making adolescents more prone to maladaptive coping strategies such as smoking or drinking to manage distress [37]. Simultaneously, childhood family violence may also coincide with social environments characterized by greater peer modeling and easier access to substances [38]. Notably, we found that peer smoking and product availability were among the factors most strongly associated with substance use, underscoring how environmental exposure can potentiate the risks conferred by early maltreatment [39,40]. In addition, parental smoking and alcohol use may further reinforce these pathways by normalizing substance-related behaviors and increasing adolescents’ exposure and access within the home environment, thereby amplifying the likelihood of initiation and continuation of use [41,42].

### 4.3. Sociocultural Context and Implications for Prevention

The sociocultural context of China may further influence these dynamics. In many low-resource and rural families, authoritarian parenting and verbal chastisement remain socially accepted, often perceived as educational rather than abusive [43]. This normalization may obscure the psychological harm of nonphysical violence and limit access to formal or informal support [44]. Rapid urbanization and large-scale parental migration have created a substantial population of left-behind children, who experience higher levels of emotional neglect and reduced supervision [45]. Previous research among Chinese adolescents has associated parental migration with elevated health-risk behaviors, including tobacco and alcohol use [46]. Evidence from rural Chinese adolescents has also linked childhood maltreatment with smoking and drinking trajectories across adolescence [1]. These findings indicate that future prevention research in China may benefit from considering emotional and verbal maltreatment alongside physical abuse and from evaluating culturally sensitive positive parenting programs.

From a policy and practice perspective, our findings highlight several areas that warrant further evaluation within multi-level prevention strategies. Child protection and parenting programs may benefit from addressing emotional maltreatment and promoting supportive communication. Previous evidence suggests that parenting programs can contribute to reductions in child maltreatment and related risk factors [44]. Schools and primary health services could evaluate trauma-informed screening and early psychological support for adolescents exposed to family adversity. In parallel, stronger enforcement of age restrictions on tobacco and e-cigarettes, combined with peer-focused prevention initiatives, may help address environmental correlates of substance use. This is particularly relevant in China, where tobacco control measures have gradually strengthened, but important challenges remain, including the regulation of youth access to e-cigarettes [18,19]. Prospective and implementation studies are needed to evaluate the effectiveness of these approaches in China and similar settings.

### 4.4. Strengths and Limitations

This study has several notable strengths. The exceptionally large, population-based sample enhances statistical power and external validity, while the ability to adjust for a broad range of environmental and socioeconomic covariates strengthens internal validity. Importantly, this work provides rare, population-level evidence from China, where childhood family violence and cumulative adversity have historically received limited empirical attention.

Nevertheless, several limitations warrant consideration. First, the cross-sectional design precludes causal inference and does not establish the temporal ordering of childhood family violence exposure and substance use. Bidirectional or shared-cause explanations, therefore, remain possible. Second, reliance on adolescents’ self-reported data may introduce recall, reporting, or social desirability bias, particularly for sensitive experiences such as maltreatment and substance use. Third, residual confounding cannot be excluded. Although the analyses accounted for multiple demographic, socioeconomic, and environmental factors, the survey did not capture several potentially relevant variables, including parental alcohol or other substance misuse, parental mental health, genetic or familial predisposition, and adolescents’ unmeasured psychiatric symptoms. Accordingly, the observed associations should not be interpreted as independent causal effects of childhood family violence. Finally, as data were collected in a single city, the findings may not generalize to regions with different cultural, socioeconomic, or regulatory contexts. Future longitudinal studies are needed to clarify temporal relationships, investigate potential mechanisms, and evaluate culturally adapted prevention strategies.

## 5. Conclusions

Childhood exposure to family violence, particularly verbal insults and emotional neglect, was significantly and cumulatively associated with adolescent tobacco, e-cigarette, and alcohol use in this large school-based sample in China. These findings suggest the importance of broadening current prevention frameworks beyond physical abuse to include greater attention to emotional maltreatment, peer influences, and access to addictive substances. Future longitudinal studies are needed to establish temporal relationships and elucidate underlying mechanisms, and well-designed intervention research is warranted to evaluate the effectiveness of multi-level, trauma-informed approaches involving schools, families, and regulatory systems as potential strategies for preventing early substance use.

## Figures and Tables

**Table 1:** Descriptive statistics of participants (N = 41,146).

Characteristic	Number of Participants	Mean (SD)/ Percent
**Age, years**	41,146	14.8 (1.6)
**Gender (%)**		
Female	21,283	51.7%
Male	19,863	48.3%
**Grade (%)**		
Primary school and Junior high school	26,663	64.8%
High school	14,483	35.2%
**Residence (%)**		
Urban	22,025	53.5%
Rural	19,121	46.5%
**Only child (%)**		
Only child	3675	8.9%
Not only child	37,471	91.1%
**Parents’ education level (%)**		
Less than high school	28,024	68.1%
High school graduate	9103	22.1%
College or above	4019	9.8%
**Social economic status ^α^**	41,146	5.03 (1.84)

**^α^** Note: Social economic status (SES) question (if each family’s position in society in terms of income, education, and job respect can be categorized into 10 classes, where 1 represents the lowest class and 10 represents the highest class, which class do you think your family is in the society?).

**Table 2:** Childhood Family Violence Exposure Among Participants (N = 41,146).

Characteristic	Number of Participants	Percent
**Childhood family violence**		
Any type of childhood family violence	10,316	25.1%
Physical violence	3207	7.4%
Physical neglect	1545	3.8%
Verbal insults	7649	18.6%
Emotional neglect	6691	16.3%
Sexual violence	804	2.0%
**Cumulative childhood family violence experiences**		
Never experienced childhood family violence	30,830	74.9%
Experienced one type of childhood family violence	4807	11.7%
Experienced two types of childhood family violence	3120	7.6%
Experienced three or more types of childhood family violence	2389	5.8%

**Table 3:** Substance Use and Environmental Risk Factors Among Participants (N = 41,146).

Characteristic	Number of Participants	Percent
**Substance use**		
Any type of substance use	8115	19.7%
Tobacco use	4299	10.4%
Electronic cigarettes use	1946	4.7%
Alcohol use	5628	13.7%
**Cumulative substance use**		
Only tobacco use	1667	4.1%
Only electronic cigarettes use	260	0.6%
Only alcohol use	3276	8.0%
Tobacco use and electronic cigarettes use	560	1.4%
Tobacco use and alcohol use	1226	3.0%
Electronic cigarettes use and alcohol use	280	0.7%
Exposure to three substance use	846	2.1%
**Environmental risk factors for Substance Use**		
Access to tobacco products	2388	5.8%
Exposure to father tobacco use	24,032	58.4%
Exposure to mother tobacco use	1402	1.4%
Exposure to teacher tobacco use	784	0.8%
Exposure to friend tobacco use	1184	1.1%

**Table 4:** Multivariable Logistic Regression for the Association Between Childhood Family Violence and Tobacco Use (n = 41,146).

Variables	Model 1			Model 2			Model 3		
	OR	95% CI	*p*	OR	95% CI	*p*	OR	95% CI	*p*
Childhood Family Violence	1.11	(1.10, 1.12)	<0.001	1.11	(1.10, 1.13)	<0.001	1.09	(1.08, 1.10)	<0.001
Age				1.13	(1.09, 1.17)	<0.001	1.10	(1.06, 1.13)	<0.001
Gender				1.88	(1.76, 2.01)	<0.001	1.50	(1.40, 1.61)	<0.001
Grade				0.90	(0.81, 1.00)	0.058	0.99	(0.88, 1.10)	0.790
Place				1.00	(0.94, 1.07)	0.955	1.02	(0.96, 1.10)	0.497
One-child status				0.97	(0.87, 1.08)	0.528	1.03	(0.92, 1.16)	0.610
SES				0.93	(0.91, 0.95)	<0.001	0.94	(0.92, 0.96)	<0.001
Parents’ education level									
High school graduate				0.92	(0.85, 1.00)	0.052	0.93	(0.85, 1.01)	0.104
College or above				0.98	(0.92, 1.06)	0.670	0.98	(0.90, 1.05)	0.535
Access to tobacco							4.16	(3.77, 4.59)	<0.001
Father Smoking							1.41	(1.31, 1.52)	<0.001
Mother Smoking							1.22	(0.99, 1.50)	0.069
Teacher Smoking							1.15	(1.05, 1.25)	0.002
Friend Smoking							4.15	(3.81, 4.52)	<0.001
AIC	27,247.44			26,572.24			23,984.06		
BIC	27,264.69			26,658.45			24,113.38		

**Note:** All categorical variables were entered as dummy variables with the following reference categories: female (Gender), primary/junior high school (Grade), rural (Residence), only child (Only-child status), and “No” for tobacco access and smoking exposure variables (father/mother/teacher/friend smoking). Parents’ education was included as a categorical variable coded as 0 = less than high school, 1 = high school graduate, and 2 = college or above, with less than high school as the reference category. SES: Social economic status.

**Table 5:** Multivariable Logistic Regression for the Association Between Childhood Family Violence and Electronic Cigarette Use (n = 41,146).

Variables	Model 1			Model 2			Model 3		
	OR	95% CI	*p*	OR	95% CI	*p*	OR	95% CI	*p*
Childhood Family Violence	1.11	(1.10, 1.13)	<0.001	1.11	(1.10, 1.13)	<0.001	1.09	(1.07, 1.10)	<0.001
Age				1.08	(1.03, 1.13)	0.001	1.02	(0.97, 1.07)	0.515
Gender				2.24	(2.03, 2.47)	<0.001	1.66	(1.50, 1.84)	<0.001
Grade				0.82	(0.70, 0.96)	0.015	0.92	(0.78, 1.09)	0.328
Place				1.14	(1.04, 1.26)	0.006	1.20	(1.08, 1.33)	<0.001
One-child status				0.84	(0.73, 0.98)	0.020	0.92	(0.79, 1.07)	0.269
SES				0.98	(0.95, 1.00)	0.053	0.99	(0.97, 1.01)	0.404
Parents’ education level									
High school graduate				0.98	(0.87, 1.09)	0.627	0.99	(0.88, 1.12)	0.919
College or above				1.02	(0.92, 1.13)	0.709	1.01	(0.91, 1.13)	0.813
Access to tobacco							4.26	(3.77, 4.81)	<0.001
Father Smoking							1.25	(1.13, 1.39)	<0.001
Mother Smoking							1.45	(1.13, 1.84)	0.003
Teacher Smoking							1.02	(0.90, 1.14)	0.797
Friend Smoking							5.64	(5.05, 6.30)	<0.001
AIC	15,383.13			15,144.72			13,159.84		
BIC	15,500.37			15,230.94			13,289.15		

**Note:** All categorical variables were entered as dummy variables with the following reference categories: female (Gender), primary/junior high school (Grade), rural (Residence), only child (Only-child status), and “No” for tobacco access and smoking exposure variables (father/mother/teacher/friend smoking). Parents’ education was included as a categorical variable coded as 0 = less than high school, 1 = high school graduate, and 2 = college or above, with less than high school as the reference category. SES: Social economic status.

**Table 6:** Multivariable Logistic Regression for the Association Between Childhood Family Violence and Alcohol Use (n = 41,146).

Variables	Model 1			Model 2			Model 3		
	OR	95% CI	*p*	OR	95% CI	*p*	OR	95% CI	*p*
Childhood Family Violence	1.13	(1.11, 1.14)	<0.001	1.13	(1.12, 1.14)	<0.001	1.11	(1.10, 1.12)	<0.001
Age				1.14	(1.11, 1.18)	<0.001	1.12	(1.08, 1.15)	<0.001
Gender				1.38	(1.30, 1.46)	<0.001	1.13	(1.06, 1.20)	<0.001
Grade				1.04	(0.95, 1.15)	0.384	1.12	(1.01, 1.23)	0.034
Place				1.23	(1.16, 1.30)	<0.001	1.28	(1.20, 1.36)	<0.001
One-child status				0.96	(0.88, 1.06)	0.444	0.99	(0.90, 1.10)	0.897
SES				0.94	(0.93, 0.96)	<0.001	0.95	(0.94, 0.97)	<0.001
Parents’ education level									
High school graduate				1.09	(1.01, 1.16)	0.021	1.12	(1.04, 1.20)	0.003
College or above				0.97	(0.91, 1.03)	0.365	0.96	(0.90, 1.03)	0.261
Access to tobacco							2.45	(2.22, 2.70)	<0.001
Father Smoking							1.38	(1.29, 1.47)	<0.001
Mother Smoking							0.84	(0.68, 1.03)	0.087
Teacher Smoking							1.20	(1.11, 1.30)	<0.001
Friend Smoking							3.98	(3.67, 4.31)	<0.001
AIC	32,379.35			31,767.13			29,705.16		
BIC	32,396.60			31,855.34			29,834.48		

**Note:** All categorical variables were entered as dummy variables with the following reference categories: female (Gender), primary/junior high school (Grade), rural (Residence), only child (Only-child status), and “No” for tobacco access and smoking exposure variables (father/mother/teacher/friend smoking). Parents’ education was included as a categorical variable coded as 0 = less than high school, 1 = high school graduate, and 2 = college or above, with less than high school as the reference category. SES: Social economic status.

**Table 7:** Adjusted Odds Ratios of Substance Use among Adolescents with Childhood Family Violence.

Variables	Tobacco Use			Electronic Cigarettes Use			Alcohol Use		
	OR	95% CI	*p*	OR	95% CI	*p*	OR	95% CI	*p*-Value
Physical violence	1.35	(1.27, 1.43)	<0.001	1.34	(1.24, 1.45)	<0.001	1.42	(1.35, 1.50)	<0.001
Physical neglect	1.17	(1.09, 1.25)	<0.001	1.25	(1.14, 1.36)	<0.001	1.26	(1.19, 1.33)	<0.001
Verbal insults	1.44	(1.39, 1.50)	<0.001	1.43	(1.24, 1.62)	<0.001	1.51	(1.46, 1.57)	<0.001
Emotional neglect	1.43	(1.37, 1.49)	<0.001	1.38	(1.30, 1.46)	<0.001	1.52	(1.47, 1.58)	<0.001
Sexual violence	1.07	(0.99, 1.16)	0.086	1.19	(1.08, 1.31)	<0.001	1.14	(1.06, 1.22)	<0.001

**Note:** All models controlled for age, gender, grade, residence, only-child status, socioeconomic status (SES), parents’ education level, access to tobacco products, peer smoking, and parental smoking.

**Table 8:** Adjusted Odds Ratios of Substance Use among Participants with Cumulative Childhood Family Violence.

Cumulative Childhood Family Violence	Smoker vs. Non-Smoker				E-Cigarette Use vs. Non-E-Cigarette Use				Alcohol Use vs. Non-Alcohol Use			
	OR	(95% CI)		*p*-Value	OR	(95% CI)		*p*-Value	OR	(95% CI)		*p*-Value
Never experienced	1	[reference]		NA	1	[reference]		NA	1	[reference]		NA
Experienced one type of family violence	2.26	2.06	2.47	<0.001	2.07	1.81	2.36	<0.001	2.34	2.16	2.54	<0.001
Experienced two types of family violence	2.95	2.66	3.27	<0.001	2.90	2.51	3.35	<0.001	3.18	2.90	3.48	<0.001
Experienced three or more types of family violence	3.81	3.42	4.23	<0.001	3.90	3.39	4.48	<0.001	3.95	3.59	4.35	<0.001

**Note:** All models controlled for age, gender, grade, residence, only-child status, socioeconomic status (SES), parents’ education level, access to tobacco products, peer smoking, and parental smoking.

## Data Availability

All inquiries regarding data access should be directed to the corresponding authors. Data sharing is limited for the public based on the Data Usage Agreement.

## Abbreviations

The following abbreviations are used in this manuscript:

ACE	adverse childhood experience
SES	socioeconomic status
SD	standard deviation
AIC	Akaike Information Criterion
BIC	Bayesian Information Criterion
CI	confidence interval
OR	odds ratio
OR	adjusted odds ratio
AUDIT	Alcohol Use Disorders Identification Test
GYTS	Global Youth Tobacco Survey
HPA	hypothalamic–pituitary–adrenal
